# The Combined Diffusion and Adsorption Concept for Prediction of Nanoparticles Transport through Dermal Layers Based on Experiments in Membranes

**DOI:** 10.3390/ijms23126419

**Published:** 2022-06-08

**Authors:** Mariola M. Błaszczyk, Jerzy Sęk, Łukasz Przybysz

**Affiliations:** 1 Department of Chemical Engineering, Faculty of Process and Environmental Engineering, Lodz University of Technology, 213 Wolczanska St., 90-924 Lodz, Poland; jerzy.sek@p.lodz.pl (J.S.); luk.przybysz@o2.pl (Ł.P.); 2 Department of Refrigeration Technology and Technique in Lodz, Institute of Agriculture and Food Biotechnology, 84 Al. Marszałka J. Piłsudskiego, 92-202 Lodz, Poland

**Keywords:** nanoparticles, drug carriers, drug delivery, microfluidics, nanoparticle diffusion, nanoparticle adsorption, membranes

## Abstract

The non-invasive introduction of active substances into the human body is a top challenge for researchers in medicine, pharmacology, and cosmetology. Development of nanotechnology and possibilities of creating more and more complex drug carriers on a nanoscale give a more realistic prospect of meeting this challenge. However, in the absence of sufficient knowledge of the mechanisms of such systems’ transport through the human skin structure, it is necessary to look deeper into these issues. There are several models describing nanoparticles transport through the skin, but they are mainly based on diffusion process analysis. In this work, a model was proposed to predict nanoparticles transport through the skin, based on the combined diffusion and adsorption concept. This approach was based on experimental studies of silver and copper nanoparticles’ diffusion process through different filtration membrane layers. Dependence of the degree of adsorption on the surface parameter was described using modified Langmuir equation. Then, these considerations were related to the structure of the stratum corneum, which made it possible to predict the changes in the mass of penetrating nanoparticles as a function of transport path length. A discussion of the presented model, depending on such parameters as nanoparticle size, skin cell thickness, or viscosity of the “intercellular cement”, was also performed.

## 1. Introduction

The ultimate goal of development is to work toward a world deprived of suffering and to provide the greatest comfort of life for every human being. In such a world, the treatment of diseases should be effective and painless. To make this possible, therapeutic substances should be delivered non-invasively, without needles and syringes. This is of particular importance when administering vaccines for various incurable diseases, as studies [1,2] report that over twenty percent of the population exhibit an inborn fear of needles, which, in many cases, result in conscious avoidance of vaccinations. Additionally, the introduction of drugs by traditional methods can become a cause of cross-infection, which carries a direct threat to human life.

It should be remembered that nowadays health is understood as not only the absence of disease but also vitality and youthful appearance. People are increasingly looking for effective pharmaceutical and cosmetic preparations that will be able to provide inhibition of, or even reduction in, the signs of aging, without surgical intervention. Cosmetic manufacturers, on the other hand, are looking for opportunities to use the latest technological advances to develop preparations that will not only act on the surface but will be able to penetrate into the deep layers of the skin and rebuild its structure there [3].

For this reason, efforts are being made to design medications that can be introduced into the human organism directly by mere contact with the body surface [4]. However, for a bioactive substance to reach inside the human body in such a way, it must penetrate the skin structure, which is a powerful barrier for most compounds [5]. Human skin consists of layers characterized by different lipophilicity, which further reduces the possibility of transport of introduced compounds. For this reason, active substances are often placed on or in the so-called carriers, and in this form, they are transported to selected skin layers [6,7]. The simplest carriers of active substances are nanoparticles, which are the matrix for many types of compounds [8,9]. The rapid progress of nanotechnology in recent years has contributed to the possibility of creating nanoparticles with well-defined parameters adapted to medical purposes [10]. However, to be able to exploit the potential offered by the use of nanoparticles as non-invasive drug carriers, it is not enough just to be able to produce them, but it is also necessary to know the mechanisms of their transport through the skin structure. The literature review also shows that the processes of adsorption or, more generally, retention of nanoparticles in particular organs of a human body are very important from the point of view of the efficiency of their introduction into organisms as carriers of therapeutic substances. This applies especially to metallic nanoparticles (MNPs) [11,12]. The role of the liver, which can retain up to 90% of nanoparticles without allowing them to move through the circulatory system, should be emphasized here. Therefore, the study of the adsorption processes of nanoparticles in the tissues of living organisms are very important to understand the role that they can actually perform as therapeutic enhancers.

**Figure 1:**
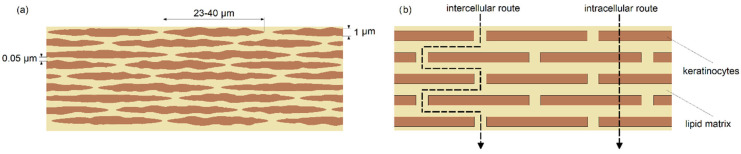
(**a**) structure of stratum corneum; (**b**) brick wall model.

Human skin consists of several layers, the most difficult to penetrate is the outer layer called the stratum corneum [13,14]. It consists of flattened cells called keratinocytes, between which there is a so-called intercellular cement (see Figure 1a). A certain brick wall model is often used to describe the transport process through dermal structures (see Figure 1b). According to this model, cells are cuboids between which there is cement [15].

Transport of substances through dermal structures can take place in three ways: through the dermal appendages; the intercellular route; and the intracellular route [16]. Dermal appendages constitute about 1% of the skin surface, therefore they do not represent an effective transport route. The intracellular route is possible only for low molecular weight compounds, which exclude many therapeutic substances. However, the most promising is the intercellular route. The movement of substances along this route is described in the literature as diffusive transport and is characterized by Fick’s first law [17,18]. The proportionality factor between the mass flux and the concentration gradient along the pathway is the diffusion coefficient. For the diffusion process of nanoparticles in liquids, the diffusion coefficient is described by the Stokes–Einstein relation [19,20], and its value is estimated to be of the order of 10^−11^ [m^2^/s] [21]. However, nanoparticles, moving in the skin, encounter skin cells on their way, which comes down to the fact that transport takes place through a specific porous structure. It is generally accepted that the transport of particles through porous structures is dependent on the ratio of the size of these particles to the size of the pores, or, in this case, the intercellular spaces (cement) [22,23,24]. That is, the smaller the diameters of the pathways by which a substance can be transported, the greater the resistance and the less intense the flow. This, therefore, suggests that for the transport of nanoparticles through dermal structures, it is the size of the intercellular spaces that have a decisive influence on their migration. In this work, the authors decided to dispute these problems. It was based on the results of a study conducted on the diffusion process of nanoparticles through filter membranes. These membranes had a mesh diameter many orders of magnitude larger than the diameter of nanoparticles. This would suggest that they will constitute a negligible barrier to the diffusion process and it would occur at a rate equivalent to the diffusion rate of nanoparticles in liquids. However, the results of the experiments indicated that the diffusion rate is significantly reduced. Based on these experiments, it was concluded that it is not the size of the space between the fibers that matters (mesh size), but the surface area of the fibers. Following this line of reasoning, the results of the experimental work were translated to modeling the diffusion process in dermal structures. On this basis, a model was developed, based on the concept of simultaneous diffusion and adsorption, describing the dynamics of nanoparticle transport through dermal layers. The presented model allows the determination of the dependence of the mass of nanoparticles that are able to pass through the structure on the thickness of this dermal structure.

## 2. Results

**Figure 2:**
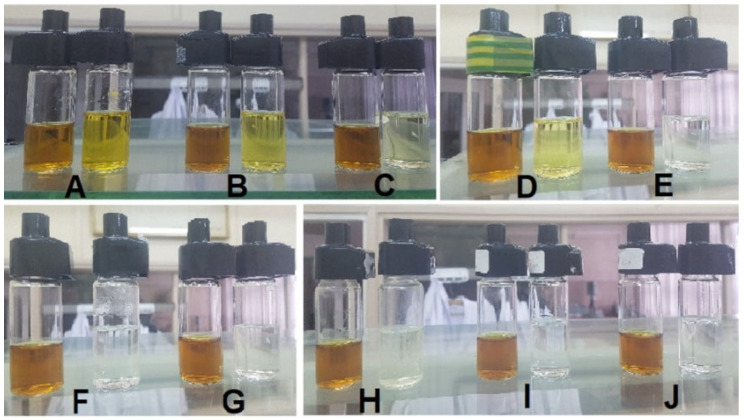
Cells with collected acceptor and donor fluid after silver nanoparticle diffusion process using different filters: (**A**)—one filter F118; (**B**)—one filter F63; (**C**)—one filter F1; (**D**)—two filters F118 and F63; (**E**)—combination of three filters F118, F63, and F1; (**F**)—two filters F1; (**G**)—three filters F1; (**H**)—one filter F9*; (**I**)—two filters F9*; (**J**)—three filters F9*.

**Figure 3:**
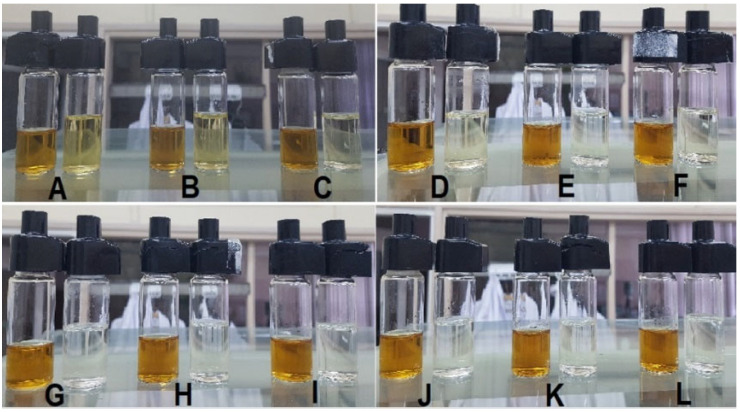
Cells with collected acceptor and donor fluid after copper nanoparticle diffusion process using different filters: (**A**)—one filter F118; (**B**)—one filter F63; (**C**)—one filter F1; (**D**)—two filters F118 and F63; (**E**)—combination of three filters F118, F63, and F1; (**F**)—two filters F1; (**G**)—three filters F1; (**H**)—one filter F9*; (**I**)—two filters F9*; (**J**)—three filters F9*; (**K**)—combination of filters in the order of F1, F9*; and F1; (**L**)—combination of filters F9*, F1, and F9*.

As described in the previous section of this work, the passive diffusion process of silver or copper nanoparticle solutions was carried out using different filter combinations. After 12 h, the liquids from the acceptor and donor chambers were poured into measuring cells and tested to determine the concentration of nanoparticles. A summary of the post-process fluids, along with the corresponding markings, is presented in Figure 2 for the nanoAg diffusion, and in Figure 3 for the nanoCu diffusion [25].

The photos presented in Figure 2 and Figure 3 show the arrangement of fluid pairs—on the left the donor fluid, on the right the acceptor fluid. As can be seen from the visual evaluation of the photos, the coloration of the acceptor fluids alone indicates the intensity of the passive diffusion process when individual filters or combinations of filters are used. When triple ones are used, it can be observed that the acceptor fluid is almost completely transparent. However, to obtain quantitative data, all fluids were subjected to conductivity tests to determine the concentration of the solutions.

**Figure 4:**
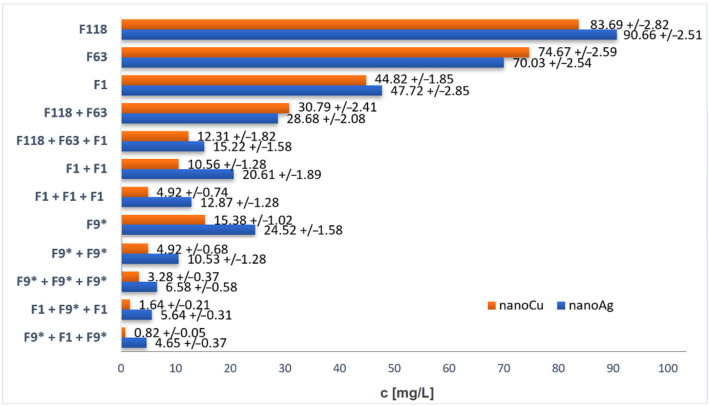
Change in concentration of acceptor fluid after nanoparticle diffusion depending on the filter used.

Based on the analysis of the concentrations of nanoparticles contained in the solutions from the acceptor chamber, it was possible to draw comparative graphs depending on the types of filters used, as shown in Figure 4.

**Figure 5:**
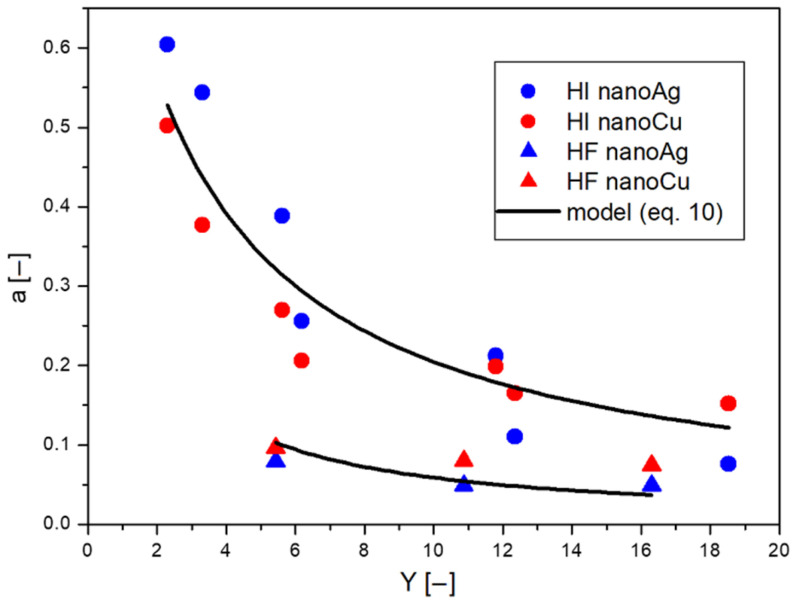
Dependence of coefficient a on surface parameter *Y* for passive diffusion process of silver and copper nanoparticles through filters with different wettability.

As can be seen from the analysis of the data in Figure 5, the largest quantity of particles entered the acceptor fluid through the 118 μm mesh size filter. However, for a filter with a mesh size almost a half smaller (63 μm), the concentration of nanoparticles did not decrease by half but only by more than 20%. When a single filter with a mesh size of 1 μm was used, relatively large results were also obtained, considering that the mesh size was reduced by as much as 118 times compared to the F118 filter, the concentration of nanoparticles in the solution was only halved. Better nanoparticle retention results were obtained when multiple layers of filters were used. Combining the two largest mesh filters (F118 and F63) resulted in a concentration of about 30 mg/L, which is 7% compared to the initial concentration of the donor fluid (400 mg/L). Adding the smallest mesh size filter reduced this value to about 4%. Very low concentrations were obtained by combining the smallest mesh filter (F1). In the case of combining two layers, the nanoparticle permeability averaged about 4%, while that of the three layers was about 2%. The use of a hydrophobic filter gave surprising results. It turned out that in the case of a single layer F9*, the concentration values obtained were less than half of the concentrations obtained when the filter with the smaller mesh size, F1, was used. Clearly, the use of the double and triple layers of the F9* filter resulted in a further decrease in concentration in the acceptor fluid. However, the highest retention was obtained with the combination of the F1 and F9* filters. Here different layering arrangements were used. An arrangement such that the hydrophobic filter (F9*) was in the center and the hydrophilic filters (F1) were on the outside resulted in slightly higher concentration values than the reverse combination. However, in both cases, it can be concluded that the concentrations obtained were so low that the filters became an impermeable barrier to the passive diffusion of the nanoparticles. Comparing the concentration values obtained for silver nanoparticles with copper nanoparticles, it can be observed that for nanoAg, in most cases the concentration values were slightly higher. This could be due to the fact that the copper nanoparticles were slightly larger than the nanoAg, which meant that the diffusion process could occur more slowly for them.

It is important to emphasize the fact that filters with mesh sizes many orders of magnitude larger than the size of the nanoparticles were used, however, despite this, the filters presented a large obstacle to nanoparticle diffusion, and, when a combination of different layers was used, an almost impassable barrier. On this basis, it can be concluded that the mesh size, and thus the size of the open spaces (channels), does not play such a big role during the process of passive diffusion through the membrane partitions. The surface area of the filter threads seems to be of greater importance, as they are the actual barrier to nanoparticles, since it is on the surface of these threads that the nanoparticles can settle. The more strands in the mesh, the larger their surface area and the more likely nanoparticles are to be deposited on them. In the case of the study presented here, the thread diameters of the filters were comparable (see Table 1); however, the denser splice of the mesh meant that the number of threads was greater for the smaller mesh filters. The use of multiple layers of filters resulted in another increase in thread area, so the surface area on which the nanoparticles could be deposited increased.

The deposition of particles on the surface of a solid is well known by the term adsorption. The concept, based on the results presented here, that nanoparticles during diffusion through the membrane also undergo some degree of adsorption, has formed the basis for further analyses aimed at describing the process of nanoparticle transport through dermal layers.

## 3. Discusion

### 3.1. Concept of Nanoparticle Transport through Partitions Based on Diffusion and Adsorption Processes

The transport of substances through skin structures is usually described in the literature by Fick’s first law, which, in its simplest form, can be written as follows:

(1) $$J=D_{ef}\frac{dc}{dx}$$

where: *J* (g/(m^2^s))—diffusing mass flux defined as the mass that diffused *m_p_* through a given cross section *A* at a given time *t*; *D_ef_*—effective diffusion coefficient (m^2^/s); *c*—diffusing substance concentration (g/m^3^), *x*—diffusion path [m].

For steady diffusion, Equation (1) can be written in the form:

(2) $$J=D_{ef}\frac{c_{o}-c_{p}}{L}$$

where: *c_0_*—is the initial concentration of the solution before the partition (g/m^3^), i.e., for the presented studies it is the initial concentration of the donor fluid (400 mg/L), *c_p_*—is the concentration of the solution behind the partition (g/m^3)^, *L*—is the partition thickness (m).

Considering the research presented in this paper, the initial concentration of the donor fluid (400 mg/L) can be taken as *c_0_; c_p_* is the concentration measured after the diffusion process in the acceptor fluid (see Figure 5), *L* is the total thickness of the filters. Knowing the diameter of the filter cross-section through which the diffusion process took place (0.02 m), it is possible to calculate its area *A* (0.000314 m^2^). Additionally, knowing the volume of liquids in which the exchange took place (*V*, 10 mL) and the duration of the process (12 h) it is possible to calculate the mass flux from the relation:

(3) $$J=\frac{m_{p}}{A\cdot t}$$

where: *m_p_* is the mass of nanoparticles that diffused through the septum from a given volume of liquid *V*, calculated on the basis of concentration, from the relation $m_{p}=c_{p}\cdot V$.

Knowing the value of mass flux *J*, calculated from Equation (3), it is possible to calculate the effective diffusion coefficient *D_ef_* from Equation (2). The results of these calculations are presented for the performed tests in Table 1.

The calculated values of the *D_ef_* diffusion coefficient indicate how fast the actual process took place. However, in order to describe what its nature is, one must first answer what factors can influence its intensity. According to the literature [19,20], the diffusion coefficient of particles in a liquid can be described by the Stokes–Einstein equation in the form:

(4) $$D=\frac{k_{B}T}{6\pi \mu d}$$

where: *k_B_* is the Boltzmann constant, *T*—temperature, *μ*—fluid viscosity, *d*—particle diameter.

Taking the data for the conditions prevailing in the experiments (*T* = 293.15 K, *μ* = 0.001 Pa∙s, *d* = 9 nm for nanoAg, *d* = 12 nm nanoCu), it is possible to calculate the coefficient *D*, which for nanoAg is 2.387∙10^−11^ (m^2^/s) while for nanoCu it is 1.79∙10^−11^ (m^2^/s). As can be seen, these values are larger than the values of the effective *D_ef_* coefficients in Table 1. This means that the actual nanoparticle transport process is delayed when there is a barrier on the diffusion path. The value of this retardation can be estimated as the ratio of the effective diffusion coefficient *D_ef_* to the coefficient *D*, according to the relation:

(5) $$a=\frac{D_{ef}}{D}$$

The values of coefficient *a* are shown for the diffusion processes studied in Table 1.

Analyzing the dynamics of nanoparticle transport in solutions, the question may arise to what degree the sedimentation of the nanoparticles affects this process. To estimate this, one can refer to the Stokes equation [26], which allows the calculation of the sedimentation velocity of a particle in a fluid, which, in the regime of laminar flow, can be described by the relation:

(6) $$u_{o}=\frac{d^{2}g\left(\rho_{s}-\rho_{c}\right)}{18\mu }$$

where: *d* is particle diameter (m), *g*—gravity acceleration (m/s^2^), *ρ_s_*—density of particles (kg/m^3^), *ρ_c_*—density of liquid (kg/m^3^), *μ*—viscosity of liquid (Pa∙s).

By substituting into Equation (8) the values for the presented studies (where *ρ_s_* for nanoAg was taken as 7874 kg/m^3^, and for nanoCu as 8950 kg/m^3^, and *ρ_c_* for water as 1000 kg/m^3^), the sedimentation velocity for nanoAg is 3.034∙10^−10^ m/s, and for nanoCu 6.239∙10^−10^ m/s. Knowing the time *t*, in which the process took place, one can calculate the distance covered by the sedimenting particle ($s=u_{0}t$). For the analyzed processes, it is 13.1 μm for nanoAg and 26.6 μm for nanoCu. Taking into account that the diffusion path *L* (resulting from the thickness of the applied filters—see Table 1) is at least 47.9 μm (for a single filter F9*) and is greater than the particles would overcome during sedimentation, this means that the nanoparticles were not able to overcome the analyzed barriers on the sedimentation path, so this process can be excluded as not relevant to the considered transport phenomena.

Based on the above discussion, it can be concluded that the movement process of nanoparticles can only be affected by the presence of an existing barrier. Thus, coefficient *a* is a quantity that describes the effect of the barrier on the diffusion process. As observed during the study of passive diffusion through filters, it is not the mesh size of the filter that is most important for the retention of nanoparticles, but its total surface area. To determine this surface area, the parameter *Y* was calculated (see Section 4 of this work), which, for the application of several filters, represented the summed *Y* values corresponding to the individual filters (see Table 1). Considering the relationship between coefficient *a* and parameter *Y*, this relationship is shown in Figure 5. As can be observed, as the area parameter *Y* increases, the value of coefficient *a* decreases. However, the coefficient a takes higher values for diffusion through hydrophilic (HI) filters than hydrophobic filters for the same *Y* values.

Based on experimental studies, it was found that the factor that diminishes the passive diffusion is the total surface area of the threads in the filter, since nanoparticles can be deposited on this surface. This deposition can be identified with the adsorption process. The dependence of the magnitude of this phenomenon on concentration or pressure (in the case of gases) is described in the literature by adsorption curves. One of the well-known adsorption curves is the Langmuir isotherm equation [27], based on which the magnitude of the actual adsorption a_d_ can be determined from the relationship:

(7) $$a_{d}=a_{m}\frac{K\cdot p}{1+K\cdot p}$$

where: *a_m_*—adsorption rate corresponding to complete filling of the monolayer, *K*—adsorption equilibrium constant, *p*—pressure or concentration of adsorbate.

According to the adsorption theory, the adsorbate concentration or pressure can be directly related to the number of sites on the surface covered by the molecules. This means that the variable in the equation can be the surface parameter *Y*. If we assume that *a_m_* is 1, and given that for our study it is important what fraction of the nanoparticles has passed through the membrane and not deposited on it, we can write the relation:

(8) $$a=1-\frac{k\cdot Y}{1+k\cdot Y}$$

where: *k* is the model constant.

Using Equation (8), the experimental data presented in Figure 5 were described for both silver and copper nanoparticles, but separately for the hydrophilic and hydrophobic filters. The description of the experimental data is presented by the black curve in the graph (see Figure 5). The constant *k* for hydrophilic filters was 0.3884, while for hydrophobic filters it was 1.6016. The course of these curves was determined by the regression method using the Microcalc Origin program.

Using the presented concept, it is possible to determine the mass that has moved by passive diffusion through a given barrier. With the knowledge of the geometric structure of a given partition, it is possible to calculate the *Y* parameter. Then, knowing the constant *k* (determined by separate studies), it is possible, using Equation (8), to calculate the coefficient *a*. Knowing the diameter of the nanoparticles *d* and the conditions under which the process took place (temperature *T*, liquid viscosity *μ*), it is possible to calculate the value of the diffusion coefficient *D* from Equation (4). This, in turn, makes it possible to calculate the *D_ef_* coefficient using Equation (5). Assuming a given initial concentration *c_0_*, the volume from which the mass exchange takes place, *V*, and the diffusion process running through a given surface, *A*, at a given time *t* through a partition of length, *L*, it is possible, using Equations (2) and (3) and knowing that *c = m_p_/V*, by performing appropriate transformations, to determine the mass of nanoparticles that diffused, from the relation:

(9) $$m_{p}=\frac{D_{ef}\cdot c_{0}}{\left(\frac{L}{A\cdot t}+\frac{D_{ef}}{V}\right)}$$

The starting point for using the presented concept is the knowledge of the constant *k*. It can be determined experimentally. Knowing the diffusion coefficient *D_ef_* for given tests, it is possible to determine the coefficient *a* (Equation (4)). Knowing the geometrical dimensions of the barrier it is also possible to calculate the *Y* parameter (equation 1). With these data, it is possible in turn to calculate the constant *k* from Equation (8). Due to the fact that the size of *k* is constant for the surface parameter *Y*, it is possible to follow how the mass of diffused nanoparticles (calculated from equation 9) will change depending on parameter *Y*. In other words, by having a single measurement of the diffusion process through a layer of a given thickness, it is possible to track how the mass of nanoparticles passing through a given number of layers will change.

### 3.2. Application of the Combined Diffusion and Adsorption Concept to Describe the Nanoparticles Transport through Dermal Structures

**Figure 6:**
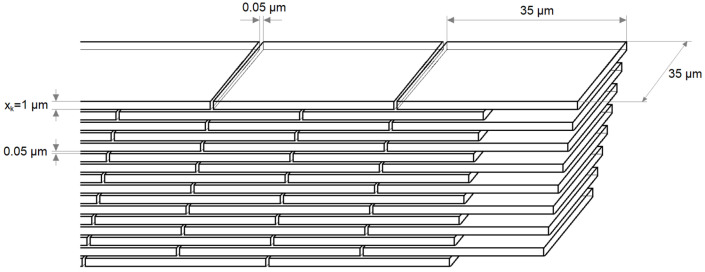
Brick structure.

The concept presented in the previous section, based on the experimental results of passive diffusion through membranes, was used to describe the transport of nanoparticles, which can be drug carriers, through dermal structures. As a first step, it is necessary to determine the surface parameter *Y*. For this purpose, it is important to look at the skin structure. Let us consider the outer layer, the stratum corneum, whose structure is presented in Figure 1a. Assuming a brick wall model (Figure 1b), it is possible to recalculate the surface parameter *Y*. Taking into account the size of keratinocytes in the stratum corneum, it can be assumed that a single brick will have dimensions of 35 × 35 × 1 μm^3^, while the space between bricks has a thickness of 0.05 μm, as shown in Figure 6.

In this arrangement, the total area *A_S_* of one brick (sum of the areas of all sides) is 2590 μm^2^. Assuming a given surface area, for example, *A_F_* = 1 cm^2^, it is possible to determine the number of bricks spaced apart by the thickness of the intercellular cement (0.05 μm) in a single layer. To do this, one first calculates how many tiles will fit over a length of 1 cm (n_d_ = 10,000 μm/(35 μm + 0.05 μm) = 285). Therefore, for an area of 1 cm^2^, the number of tiles will be *n = n_d_^2^* = 81,400. Multiplying now the number of tiles by the area of a single tile *A_S_*, we get the summed value of the area of all of the bricks for the entire single layer. Then, knowing this value, it is possible to calculate the surface parameter *Y*, according to Equation (1), which for a single layer is 2.108.

In the application of the presented concept, it is necessary to know the constant *k*. To determine it, the effective diffusion coefficient *D_ef_*, obtained from experimental studies on the transport of nanoparticles through a barrier imitating a skin structure, should be available. The geometry of this partition should be recalculated to obtain the *Y* parameter—according to the procedure presented above, but referring to a specific structure taking into account the actual number of layers. Knowing the dimensions of the nanoparticles, it is possible to calculate the coefficient *D* (Equation (4)). Then, the coefficient *a* must be calculated (Equation (5)), which in turn makes it possible to determine the constant *k* (Equation (8)). However, for modeling purposes, in further calculations, it was assumed that constant *k* is the same as the results of the presented studies, and is 0.3884 for hydrophilic structures and 1.6016 for hydrophobic structures.

By having a constant *k*, either assumed or obtained from single experimental studies, for the diffusion process of nanoparticles through a structure consisting of a specific number of single layers, it is possible to predict how the diffusion process will occur at a different, arbitrarily given number of single layers. In other words, it is possible to determine what will be the mass of nanoparticles that is able to diffuse through the skin depending on the thickness of this structure *L*. It is necessary, for each given thickness of the skin structure *L*, to calculate the parameter *Y* by summing this parameter for a single layer. The thickness of a single layer *x* is calculated as the sum of the cell thickness (*x_k_* = 1 μm) and the cement thickness (*x_c_* = 0.05 μm). With parameter *Y* (for given thickness) and constant *k*, it is possible to calculate coefficient *a* (for given thickness) (Equation (8)). Assuming the set nanoparticle size *d* and given process conditions (temperature, liquid viscosity), the value of the *D* coefficient can be calculated (Equation (6)) and in the next step—*D_ef_* coefficient (Equation (5)). Assuming the process conditions (*A, V, c_0_, t*) it is possible to calculate the mass of nanoparticles passing through the layer (Equation (11)). This allows us to obtain the dependence of the mass *m_p_* on the layer thickness *L*.

**Figure 7:**
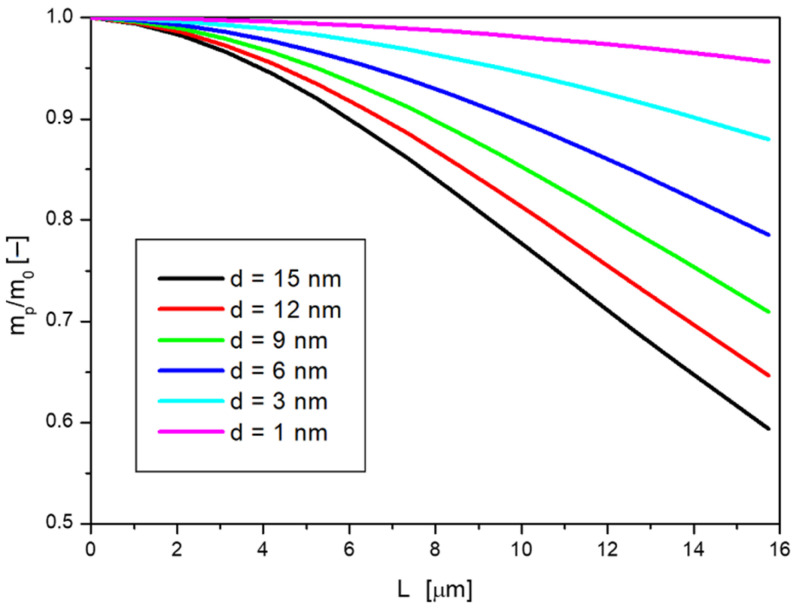
Dependence of dimensionless nanoparticle mass on skin film thickness for different particle diameters.

For the results presented here, the mass exchange area *A* was assumed to be 1 cm^2^, the transfer occurs from a volume *V* equal to 0.2 cm^3^ (which is the same as a layer of solution over the skin structure with an area of 1 cm^2^ and thickness of 2 mm), the initial concentration of nanoparticles *c_0_* is 10 g/L. Other process conditions were assumed as they were during the tests for diffusion through membranes (Section 2 and Section 4 of this work). By performing the subsequent calculation steps with the procedure outlined above, it was possible to calculate the dependence of the dimensionless mass of the passing nanoparticles (calculated as the ratio of the mass of passing nanoparticles *m_p_* to the initial mass of nanoparticles *m_0_*) on the layer thickness *L*, for different nanoparticle sizes *d*, as presented in Figure 7.

By following the changes in the particle mass that is able to diffuse at a given layer thickness for different sizes of nanoparticles, it can be observed that the larger the diameter *d*, the smaller the mass passing *m_p_* at a given value of *L*. This is consistent with the general concept of diffusion, where particles with larger sizes diffuse more slowly. For small particles, the skin barrier, will not be as large, so their mass at greater depths will be large. In this case, a fifteen-fold increase in size resulted in a decrease in their mass by about 40% at a depth of about 15 μm. With the concept adopted, it is also possible to determine the layer thickness above which no more mass passes. A limit must be adopted here, for example, for the case presented, a value of the *m_p_/m_0_* ratio below 0.005 is considered to be the one below which the process can be considered not to occur. For the diffusion of nanoparticles with diameters of 1 nm, the limiting number of layers is *N_L_* = 3300, whereas, for nanoparticles with diameters of 15 nm, the value is 860, which means that particles of size fifteen times larger have about one quarter shorter range.

**Figure 8:**
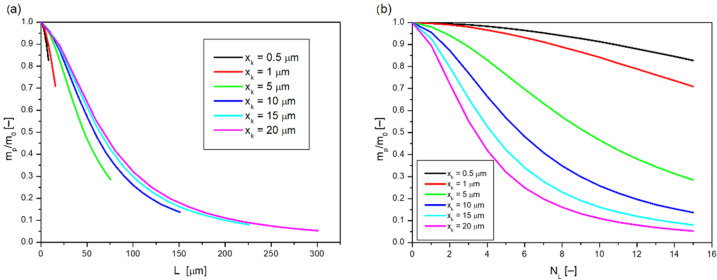
Dependence of dimensionless nanoparticle mass on (**a**) skin layer thickness; (**b**) number of individual cell layers when diffusing through skin structures with different cell sizes (bricks).

The human skin is made up of cells, the size of which can depend on many factors, mainly location on the body, age, hydration, etc. When considering the penetration of nanoparticles through the skin layers, an important parameter is the size of the cells, especially their dimension in depth *x_k_* (see Figure 6). Figure 8 shows the dependence of dimensionless nanoparticle mass, a) on skin layer thickness *L*, and b) on the number of individual cell layers *N_L_* at different dimensions *x_k_*.

By analyzing the curves in Figure 8a, it can be seen that the slope of the curves at small values of *L* is large, but at large values of layer thickness for large cell dimensions *x_k_* the dimensionless mass of nanoparticles does not change significantly. A better comparison of the variation of dimensionless nanoparticle mass as a function of cell size can be made by varying it with the number of individual *N_L_* layers (Figure 8b). When the cell dimension *x_k_* is small, their total surface area is also small, so the nanoparticles are in contact with a small area where they could be captured. As a result, the mass of nanoparticles after the 15th layer is relatively large. For large values of *x_k_*, a drastic decrease in dimensionless mass can be observed for the first layers, however, for subsequent layers, it can be observed that the decrease is smoother and smoother. The mass of nanoparticles for the 15th layer is for large values of *x_k_* = 20 μm, and is small, more than 15 times smaller than for *x_k_* = 0.5 μm.

**Figure 9:**
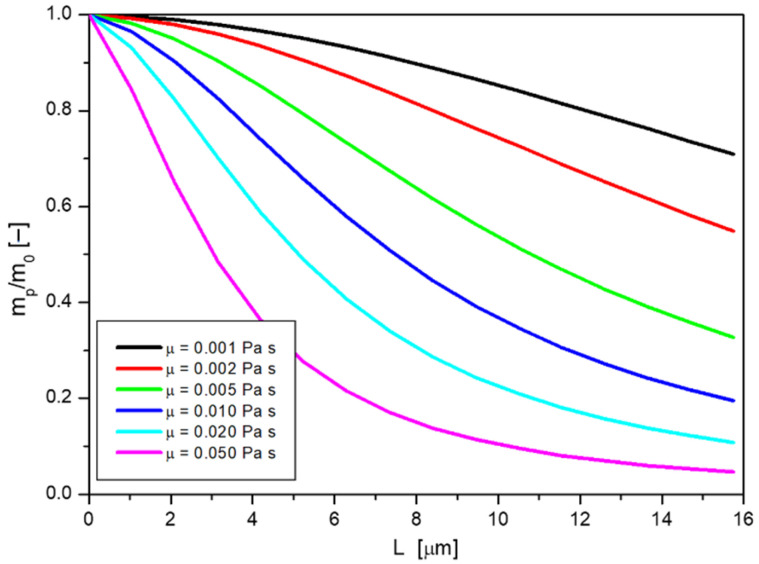
Dependence of dimensionless nanoparticle mass on skin film thickness for the diffusion of liquids with different viscosities.

The presented analysis concerns the movement of nanoparticles through the intercellular cement. This cement consists of a mixture of various substances, so its viscosity can be significant, which absolutely affects the process of transport of nanoparticles deep into the skin. Figure 9 presents the dependence of dimensionless nanoparticle mass on layer thickness *L* at different values of intercellular cement viscosity, according to the model assumptions.

As can be observed in Figure 9 the lower the viscosity the more mass is passed through a given thickness. However, at high viscosity values, a drastic decrease in the penetrating mass of nanoparticles can be observed, even at small film thicknesses. For larger values of *L*, the passing mass is small, but the decrease with depth is smoother. Assuming, as in the previous case, a limit of dimensionless mass is equal to 0.005, below which the displacement process can be considered not to occur, it can be concluded that at a viscosity of 0.001 Pa∙s this barrier occurs at 344 layers and for a viscosity of 0.05 Pa∙s it is at 48 layers.

**Figure 10:**
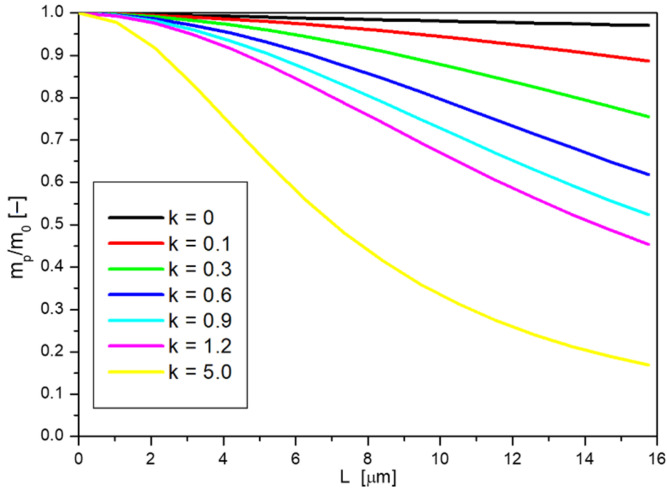
Dependence of dimensionless nanoparticle mass on skin film thickness at diffusion with different values of constant k.

For the presented examples, the constant *k* was assumed to equal 0.3884. However, according to the procedure described earlier, this constant can be determined experimentally and may take various values. In order to analyze the shape of the curves, the dependence of dimensionless mass on layer thickness was calculated, according to the assumptions of the model, for different assumed constants *k*, with the invariability of the remaining parameters, which is presented in Figure 10.

In the case where constant *k* equal to 0 was assumed, there was no retention of nanoparticles on the surface. The coefficient *a* in this situation is equal to 1 (Equation (8)), which in turn leads to *D_ef_* = *D* (Equation (5)), i.e., the effective diffusion coefficient is equal to the diffusion coefficient of nanoparticles in the liquid calculated from the Stokes–Einstein equation (Equation (4)). The dependence of the passing nanoparticle mass on the film thickness is then a straight line. When the value of the constant *k* is greater than 0, the capture (adsorption) of nanoparticles on the solid surface occurs, causing the mass *m_p_* to further decrease and the nature of the curve is no longer linear. The higher the value of the constant *k*, the more noticeable the deviation from a straight line. For very large values of the constant *k*, it can be observed that the dimensionless mass decreases strongly at small skin layer thicknesses, but at larger thicknesses, the decrease is smoother. 

It was considered that the presented model is limited to the description of processes related to pure diffusion resulting from thermal movements of molecules causing transport of nanoparticles introduced into the system. However, the model does not include processes related to convective phenomena induced by external forces such as pressure difference, pressing or stirring forces, etc., whose contribution during diffusion processes through epidermal layers is probably small. In the case of modeling such phenomena, it would be necessary to modify the model with convective components, which may become the subject of further research.

## 4. Materials and Methods

The study of the diffusion process of nanoparticles through membranes was carried out using a glass Franz diffusion chamber. This chamber consists of two parts: donor and acceptor, between which a filtration membrane was placed. The lower part of the chamber was filled with acceptor fluid, which was distilled water. The donor chamber was filled with the solution of the test substance, which was the aqueous solution of silver or copper nanoparticles. The volume of liquid in the upper and lower chambers was 10 mL. The diameter of the filter placed between the chambers was 0.02 m. The temperature at which the measurements were conducted was 22 °C. After filling particular parts of the chamber with liquids the process of passive diffusion took place for 12 h. After this time, acceptor and donor liquids were collected from the chambers and analyzed to determine the concentration of nanoparticles. In order to provide more accurate results of the diffusion process, nanoparticle diffusion studies were performed simultaneously on two Frantz chambers (to ensure the same external conditions) and were then repeated. This resulted in four samples, for each the concentration was measured, these values were averaged and their deviations were calculated.

The diffusion process involved silver (nanoAg) and copper (nanoCu) nanoparticles, which were purchased as colloidal solutions. The manufacturer of the solutions was the company Limpio FHU (Ilawa, Poland). The average diameter of nanoparticles was 9 nm for nanoAg and 12 nm for nanoCu. The concentration of nanoparticles was 4000 mg/L; however, solutions diluted ten times, i.e., with *c_0_* = 400 mg/L, were used for the study. In order to verify whether the prepared nanoparticle solutions undergo aggregation processes while studying their diffusion through the membrane, additional measurements were made. Analysis was performed on the PSA 1190 Particle Distribution Analyzer (Graz, Austria). PSA equipment through laser diffraction technology can determine the particle size and the size distribution of both liquid dispersions and dry powders. Additional nephelometric analysis was performed, using Turbican LabExpert (Toulouse, France). In this device, the measuring cell containing the sample was scanned with a light beam. Using detectors, the amount of reflected light (BS) and light transmitted (T) through the beam was measured. Scanning took place for 12 h (the same time as diffusion process proceeded), which allowed the observation of changes in the structure along the sample over time. Based on the results of these measurements, it can be concluded that both nanoAg and nanoCu solutions did not change in structure over time, indicating the absence of the nanoparticle aggregation phenomena.

The determination of the nanoparticle concentration was based on conductometric measurements. Both silver and copper are excellent conductors and their aqueous nanoparticle solutions also showed a high dependence on the nanoparticle concentration for electrical conductivity. Therefore, in the first step of the study, calibration curves of the electrical conductivity of the solutions were prepared from a known concentration of nanoparticles. Then, the conductivity readings of the obtained solutions from the post-diffusion measurements were applied to this calibration curve and the concentration was read. Conductivity measurements were performed using a CC-401 conductivity meter from Elmetron, equipped with a Hydromet ERH-AQ1 probe.

**Figure 11:**
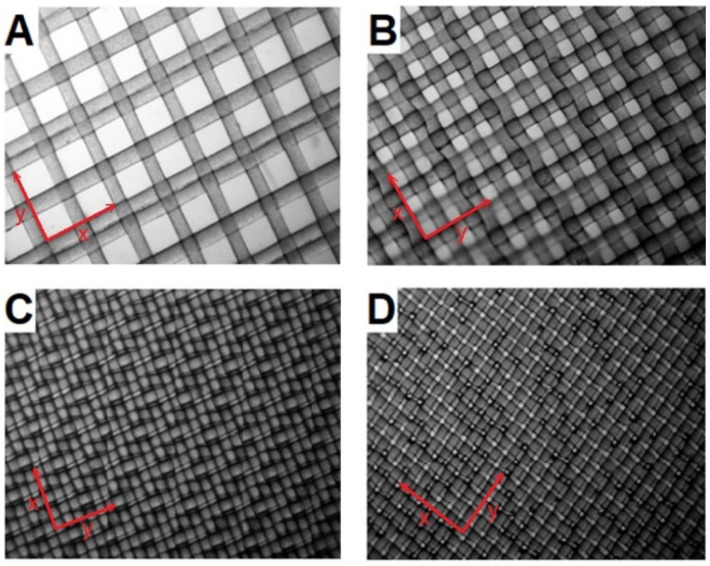
Hydrophilic filters with mesh sizes of 118 μm (**A**); 63 μm (**B**); 1 μm (**C**); and hydrophobic 9 μm filter (**D**) at 200× magnification.

Four types of commercially available filters, manufactured by SEFAR AG (Heiden, Switzerland) and made of polyacrylamide, were used in this study. Three of the filters used were hydrophilic (HI), while one was surface modified and exhibited hydrophobic (HF) properties. The filters differed in mesh size and thread thickness. Figure 11 shows microscopic images of the filters used. Calculations of the necessary filter parameters were also made, based on the microscopic measurements. Taking into account the measured mesh size, thread thickness, splicing method, and assuming that the threads have a circular cross-section, it was possible to determine the total *A_N_* thread area for the assumed *A_F_* filter area. The ratio of these areas allowed the calculation of a dimensionless *Y* parameter, used later in this work. The *Y* parameter was thus calculated as the relation:

(10) $$Y=\frac{A_{N}}{A_{F}}$$

Assuming that the area of A_F_ is a square with side dimensions *h* = 1 cm, it is possible to calculate the area of *A_N_* threads as a product of the number of all of the threads and the area of one thread, which can be represented by the relation:

(11) $$A_{N}=\left(n_{x}+n_{y}\right)\times \pi \times d_{f}\times h\;$$

where: *n_x_*—number of horizontal threads—determined on the basis of microscope measurements, *n_y_*—number of vertical threads, *d_f_*—thread diameter

The number of threads horizontally and vertically may vary and is dependent on the splicing method. In the case of the filters used, the number of vertical threads differs from the number of horizontal threads, due to the fact that a splice is used in which double threads occur next to each other in one direction in every other splice (which can be observed in Figure 11), while in the other direction the threads occur at the same distance from each other. Knowing the thread diameter and the mesh diameter, one can count how many threads occur in both directions on the assumed *A_F_* surface. For example, assuming an *A_F_* area of 1 cm^2^, it can be assumed to be a square with side *h* = 10,000 μm. Given a filter where the mesh size was *l_h_* = 118 μm and the thread diameter is *d_f_* = 55.46 μm, considering the splice shown in Figure 11A, the number of threads can be calculated. In the direction where there are no double splices, the number of threads is 58 at this length *n_x_* ($n_{x}=h/\left(d_{f}+l_{h}\right)$), and on the length where every second double weave occurs (assuming that there is no free space between adjacent threads). This means that for three threads there are two meshes, which creates a sequence of length $h_{c}=3d_{f}+2l_{h}$, and such sequences in the considered length are $c=h/h_{c}$. Knowing that there are three strands in one string, the number of strands in the length under consideration is $n_{y}=3c$, which, after substituting the values, gives 74. Since the strands are spliced in two directions, the total number of threads is 132. In a similar way, the numbers of strands for the other filters were calculated, and from them the area of the threads and the *Y* parameter. Table 2 presents the parameters of the tested filters.

Investigations of the passive diffusion process were carried out using single filter layers as well as various combinations of double or triple layers. The combination of filters is indicated by the sum of their symbols. For example, the designation F118+63+1 indicates that a partition consisting of three layers was used, the first from the top being filter layer F118, then filter layer F63 and finally filter layer F1. The calculation of the *Y* parameter was completed by summing the *Y* parameters corresponding to each filter.

## 5. Conclusions

In this paper, an attempt was made to describe the process of diffusion of nanoparticles through skin structures. As a basis for the presented assumptions, the results of experimental studies relating to the process of diffusion of silver or copper nanoparticles through filter membranes were used. These studies were carried out through various types of filters with different mesh sizes. In addition, the transport process of nanoparticles was also studied when layers of individual filters were overlapped. The mesh sizes of the filters ranged from 1 μm–118 μm, which is at least three orders of magnitude larger than the diameters of the nanoparticles. Despite this, the filter membrane layer was an effective barrier to nanoparticle transport. Given this, it was concluded that, counterintuitively, the size of the mesh (i.e., the voids in the porous structure) is not essential for the diffusion process through the membrane. It was considered that nanoparticles passing through the barriers may undergo another process, which can be identified with the process of their adsorption on the surface. Therefore, the dependence of the mesh surface parameter *Y* on the intensity of the nanoparticle diffusion process was investigated. This dependence was described using a modified adsorption curve, for which the model constant was determined. In the next step, the presented approach was applied to modeling the diffusion of nanoparticles through skin layers. For such layers, surface parameters were also calculated and, based on the assumed model constant, the degree to which a particular skin layer structure inhibits the diffusion process was determined. This allowed us to calculate the mass of nanoparticles that was able to pass through a specific skin layer under given conditions. The discussion of the presented model was then carried out with the change of different parameters, such as nanoparticle size, skin cell size (bricks), and viscosity of the substance contained in the intercellular cement. It was also investigated how the curves of the dependence of *m_p_* on *L* will proceed at different values of the model constant *k*. In the case when the constant *k* is 0, there is no barrier, and the diffusion process will be reduced to the diffusion of nanoparticles in liquids described by the Stokes–Einstein equation.

The presented approach to describe the transport of nanoparticles through dermal structures based on the concept of diffusion combined with adsorption represents a new perspective to characterize this process. The idea is based on capturing the degree of retention of nanoparticles in a given structure not depending only on the reciprocal ratio of their size to the size of the transport pathways (intercellular cement spaces), but primarily on the size of the total surface area that the nanoparticles come in contact with during transport. With this approach, it is possible to explain why particles of a small size move with such great difficulty through porous structures with large void spaces. Using the presented concept to describe the transport of nanoparticles in skin, it is possible to determine how many of them are able to pass through a specific skin layer. This, in turn, allows the estimation of how much of the active substance can be delivered by nanocarriers to a given site. This is of immense importance for the development of non-invasive medical therapeutics.

## Figures and Tables

**Table 1:** Summary of calculations for nanoparticle diffusion.

Filter	*L* × 10^−5^ (m)	Y (−)	*D_ef_*∙10^−11^ (m^2^/s)		*a* (−)	
			nanoAg	nanoCu	nanoAg	nanoCu
F118	5.55	2.30	1.1982	1.0818	0.5020	0.6043
F63	5.75	3.31	0.9000	0.9733	0.3770	0.5436
F1	4.93	6.18	0.4924	0.4587	0.2063	0.2562
F118 + F63	11.30	5.62	0.6433	0.6947	0.2695	0.3881
F118 + F63 + F1	16.23	11.79	0.4732	0.3798	0.1983	0.2122
F1 + F1	9.86	12.35	0.3949	0.1972	0.1654	0.1102
F1 + F1 + F1	14.79	18.53	0.3625	0.1359	0.1519	0.0759
F9*	4.79	5.43	0.2308	0.1414	0.0967	0.0790
F9* F9*	9.59	10.87	0.1911	0.0881	0.0801	0.0492
F9* + F9* + F9*	14.38	16.30	0.1773	0.0877	0.0743	0.0490
F1 + F9* + F1	14.65	17.79	0.1545	0.0445	0.0647	0.0249
F9* + F1 + F9*	14.52	17.04	0.1259	0.0220	0.0528	0.0123

**Table 2:** Filter parameters.

Symbol	Wettability	Mesh Size *l_h_* (μm)	Thread Diameter *d_f_* (μm)	*Y* (−)
F118	HI	118 +/−0.10	55.46 +/−0.10	2.302
F63	HI	63 +/−0.10	57.53 +/−0.10	3.313
F9*	HF	9 +/−0.10	47.93 +/−0.10	5.434
F1	HI	1 +/−0.10	49.30 +/−0.10	6.176

* indicates that the filter was hydrophobic.

## Data Availability

Not applicable.
